# Transport of *Babesia venatorum*-infected *Ixodes ricinus *to Norway by northward migrating passerine birds

**DOI:** 10.1186/1751-0147-53-41

**Published:** 2011-06-23

**Authors:** Gunnar Hasle, Hans P Leinaas, Knut H Røed, Øivind Øines

**Affiliations:** 1 Reiseklinikken - Oslo Travel Clinic, St Olavs plass 3, 0165 Oslo, Norway; 2 Department of Biology, University of Oslo, P.O. Box 1050 Blindern, N-0316 Oslo, Norway; 3 Department of Basic Sciences and Aquatic Medicine, Norwegian School of Veterinary Science, Oslo, Norway; 4 Norwegian Veterinary Institute, P.O. Box 750 Sentrum, 0106 Oslo, Norway

## Abstract

**Background:**

Bovine babesiosis is regarded as a limited health problem for Norwegian cows, and the incidence has decreased markedly since the 1930s. Rare cases of babesiosis in splenectomised humans from infection with *Babesia divergens *and *B.venatorum *have been described. The objective of this study was to determine whether birds can introduce *Babesia*-infected ticks. There are between 30 and 85 million passerine birds that migrate to Norway every spring.

**Methods:**

Passerine birds were examined for ticks at four bird observatories along the southern Norwegian coast during the spring migrations of 2003, 2004 and 2005. The presence of *Babesia *was detected in the nymphs of *Ixodes ricinus *by real-time PCR. Positive samples were confirmed using PCR, cloning and phylogenetic analyses.

**Results:**

Of 512 ticks examined, real-time PCR revealed five to be positive (1.0%). Of these, four generated products that indicated the presence of *Babesia *spp.; each of these were confirmed to be from *Babesia venatorum *(EU1). Two of the four *B. venatorum*-positive ticks were caught from birds having an eastern migratory route (*P*< 0.001).

**Conclusions:**

Birds transport millions of ticks across the North Sea, the Skagerrak and the Kattegat every year. Thus, even with the low prevalence of *Babesia*-infected ticks, a substantial number of infected ticks will be transported into Norway each year. Therefore, there is a continuous risk for introduction of new *Babesia *spp. into areas where *I. ricinus *can survive.

## Background

Ticks have limited mobility [1], but can be transported over long distances when feeding on their vertebrate hosts. In particular, avian hosts can efficiently transport ticks across geographical barriers such as oceans and deserts [2-6].

Norway is in the northernmost range for the distribution of *Ixodes ricinus*, which is a vector for many human and animal pathogens [7]. In a review by Gorenflot [8], 83% of the 28 European cases of human babesiosis occured in splenectomised individuals, and 76% were reported to be due to *Babesia divergens*. Apicomplexans of the genus *Babesia *have greater vertebrate host specificity than other tick-borne pathogens, such as *Borrelia *spp. and *Anaplasma phagocytophilum*. None of the described *Babesia *species can have both bird and mammal hosts [9,10]. The first report of *Babesia canis *in Norway occurred recently; the tick was found on a dog that had not been abroad [11]. The main vector for *B. canis *is *Dermacentor reticulatus*, which is not native to Norway. However, a larva of *Dermacentor *sp. was recovered on a northward-migrating willow warbler (*Phylloscopus trochilus*) in Akeröya in 2005 [6]. *B. divergens*, which causes redwater disease in cattle, is widespread in Europe [12]. This pathogen was first described in Norway in 1901 [13] and has been recovered and confirmed in Norway, using molecular tools (Ø. Øines unpublished results). A comparison of the data of Thambs-Lyche from the 1930s [14] with data from the Norwegian Dairy Herd Recording System (NDHRS) indicates that the incidence of babesiosis in Norwegian cattle has decreased, although the infection remains highly prevalent in some coastal locations in Norway such as Lista (longitude E 6.7°) (47% of 34 tested cows had antibodies against *B. divergens*) and the island Jomfruland (longitude E 9.6°) (68%, N = 25) [15]. Lundsett tested 439 flagged ticks along the southern Norwegian coast (although not specifically in cow-pasturing areas) and found only a single tick that was positive for *B. divergens *by polymerase chain reaction (PCR) analysis [16]. Radzijevskaja and colleagues [17] found no *Babesia *sp. in 91 ticks (16 adults and 75 nymphs) collected from Jomfruland in Norway. There are no data to support the presence of other *Babesia *species related to *B. divergens *in Norway. However, in this study, we report the presence of *B. venatorum *(*Babesia *species, EU1) in ticks found on migratory birds. This species was first characterised by Herwaldt in 2003 [18] and has been recovered from roe deer in Slovenia [19] and France [20] and from *I. ricinus *[21,22]. Three cases documenting infection with this species in splenectomised humans [18,23] have raised concern for *B. venatorum *becoming an emerging tick-borne human disease.

The purpose of this study was to assess the prevalence of *Babesia *on ticks collected from northward migratory birds arriving in Norway and to document the potential for spread of this parasite across the sea. This is the first report finding *Babesia *on ticks from birds. In comparison, a Polish study [24] did not detect *Babesia *in 442 tick specimens collected from birds.

## Materials and methods

Four Norwegian bird observatories along 300 km of the southern Norwegian coast were involved in the study; from west to east, these included Lista, Jomfruland, Store Færder and Akerøya [6]. At Akerøya, six hours of flagging yielded five ticks, and at Store Færder, two hours of flagging did not yield any ticks. Although the yield of ticks may vary through the day and through the season, our experience is that ticks are detectable at any time during spring, summer and autumn at localities where they occur. Therefore, it is questionable whether these two islands have any local tick populations. By contrast, ticks are common on Jomfruland and on the mainland locality of Lista [6]. There are pasturing cattle in Jomfruland and Lista, but there are only pasturing sheep at Akerøya or Store Færder, and no cattle or cervid animals. Altogether, 9,768 passerine birds on northward migration were caught with mist nets during the spring migrations of 2003, 2004 and 2005. The birds were examined for ticks around the eyes, beak and ear openings. The ticks were picked off with tweezers and placed in 70% ethanol for subsequent examination. One vial was used for each bird, and tweezers were sterilised with a flame between uses.

The ticks were examined using a stereomicroscope for species identification and for estimation of feeding status. In total, 1,440 nymphs and 517 larvae were found on 713 birds. Five species of ticks were found [6]. However, only *Ixodes ricinus *was included in this study. At the sites in Jomfruland and Lista, it was necessary to minimise the inclusion of ticks from resident birds. Therefore, for these two locations, we analysed only fully or almost fully engorged tick nymphs collected from birds caught before or during the peak migratory arrival time for each species, as engorgement is not evident during the first 24 hours following attachment [25]. To avoid including unengorged specimens, only obviously thickened nymphs were recorded as engorged. Because there were very few ticks on Akerøya and Store Færder, we examined all nymphs collected from birds at these two sites for pathogens. Larvae were not included in this study, as there was considerable uncertainty as to whether the reduced amounts of *Babesia *DNA obtained from larvae, in comparison to that from nymphs, would alter the sensitivity of the test. In total, 512 ticks were selected for examination. Of these, 332 were engorged and were from all the locations and 180 were unengorged or slightly engorged and were from the two locations assumed not to have resident tick populations.

The specimens were crushed with the tip of a glass rod, which was discarded after each use, and DNA was isolated using a spin-column method, using the DNeasy Blood & Tissue Kit (Qiagen), according to the manufacturer's protocol. To avoid interspecimen contamination, the ticks were handled with tweezers that were flame-decontaminated after each use. Engorged and unengorged ticks were treated using the same methods. A contract laboratory, Telelab (now Unilabs Telelab, Skien, Norway), performed the initial screening of the samples using real-time PCR to detect *B. divergens *DNA published by Radzijevskaja et al. [17]. The PCR mix consisted of 5 μL BdiF (3 pmol/μL), 5 μL BdiR (9 pmol/μL), 2 μL BdiT (5 pmol/μL), 8 μL PCR water and 25 μL TaqMan Universal PCR Mastermix (Applied Biosystems, Foster City, CA, USA) in a total volume of 45 μL per sample, with 5 μL of sample DNA or control(dH2O). After heat activation at 93°C for 10 minutes, the two-step PCR protocol consisting of the following steps ran for 40 cycles: denaturation at 94°C for 15 seconds and annealing/extension at 60°C for 60 seconds. Signals were read using an Applied Biosystems AB7000 sequence-detection system and were calculated against a dilution series of synthetic amplicon DNA supplied by Applied Biosystems and standards of *B. divergens *DNA (30,000, 3,000, 300 and 30 DNA copies). Only signals above the threshold line (ΔRn = 1e-1) at or before cycle (*Ct*) 37 were interpreted as a positive. DNA from the five positive samples were then used for the two separate nested PCR reactions described by Zintl and collagues [26]. PCRs were performed with two positive controls (*B. divergens *and *B. gibsoni*) in each run. The multiple PCR products were cloned using One Shot^® ^TOP-10 *E.coli *TA Cloning^® ^by Invitrogen (Carlsbad, USA) and pCR^®^2.1 plasmid. Transformed bacteria were grown overnight at 37°C on LB agar plates containing 75 μg/ml ampicillin and X-gal. From each PCR sample that was cloned, 16 to 24 white colonies were selected and used as templates for a PCR reaction using M13-reverse and T7 primers. Several of these PCR products were sequenced using these primers at Macrogen Europe (http://www.macrogen.com). Chromatograms were edited, trimmed for vector contamination and assembled using Contig Express in Vector Nti Advance^® ^11.5 (Invitrogen, Carlsbad, USA). An initial BLASTN search (http://blast.ncbi.nlm.nih.gov/Blast.cgi) for several of the sequence assemblages made in Contig Express revealed matches for sequences from *Ixodes*, uncultured eucaryotes and other non-*Babesia *targets. However, many of the sequence assemblages produced matches for *Babesia*. Cloned sequences from one of the five samples did not match any *Babesia *entries in BLASTN searches even after repeated PCR and cloning. The remaining four samples, however, contained cloned sequences matching *Babesia *18S DNA from the databases. Each of the nested PCR products were assembled using high-quality chromatograms into a single sequence. Sequences extending beyond the nested PCR primers were deleted to avoid any confounding errors introduced by the primers. The two nested PCRs reported by Zintl and colleagues [26] target two regions of 18S DNA in separate PCRs (5' and the 3') and produce two non-overlapping sequences due to deletion of regions protruding from the annealing sites of the primers. The 472 bp 5' and 534 bp 3' sequences from each clone were found to contain no more than 3 nucleotide ambiguities (substitutions, deletions or polymorphisms) when compared to the sequence of *Babesia *EU1 GQ888709. This corresponded to a maximum difference of 0.6% from this 'backbone' sequence.

It is possible that the infrequent substitutions found in the individual chromatogrammes were due to errors from the Taq-polymerase or basecalling or that they may represent intra-host nucleotide differences from *Babesia *present in each sample. However, when applying the principle of the majority rule to the chromatograms, these substitutions would be eliminated hence producing sequences identical to the GQ888709 Genbank entry for *B. venatorum *(EU1). The two non-overlapping sequences from the separate nested PCR's were included in an alignment that consisted of 18S sequences from each of the following *Babesia *species: *B. gibsoni *(AB118032), *B. odocolei *(AY237638), *B. divergens *(AY572456), *B. capreoli *(AY726009), *B. canis canis *(AY072926), *B canis vogeli *(AY371198), *B. canis rossi *(DQ111760), *B. kiwiensis *(EF551335), *B occultans *(EU376017), *B. bovis *(AY150059), *B. equi *(AY150062), *B. caballi *(AY309955), *B. motasi *(AY533147), *B. coco *(AY618928) and two sequences from *Babesia venatorum *(EU1) (GQ888709 and HQ830266). The alignment was exported to MEGA 5 [27], and a Neighbor-joining tree was constructed using 500 bootstrap replicates with pairwise deletion of gaps and missing data. The nucleotides in positions 505-992 and 1090-1626, relative to the complete alignment, were included in a single phylogenetic analysis which used the sequence data produced by the two nested PCRs.

The National Board of Animal Experimentation approved the field collecting and handling of the birds.

## Results

Thirty-three species of birds carrying *I. ricinus *nymphs were collected. Of the 512 nymphs investigated, 114 (22.2%), 98 (19.1%), 54 (10.5%), 40 (7.8%), 38 (7.4%), 31 (6.1%), 23 (4.5%), 22 (4.3%) and 14 (2.7%) were found on *Erithacus rubecula, Turdus merula, Turdus philomelos, Phoenicurus phoenicurus, Prunella modolaris, Phylloscopus trochilus, Sylvia curruca, Turdus iliacus *and *Sylvia atricapilla*, respectively. Only two ticks were found on *Luscinia svecica *and one on *Phylloscopus trochiloides*, and these are the only bird species in this material that have an eastern migratory route. Initial real time analysis revealed five specimens to be positive for the presence of *Babesia *sp. Subsequent PCR and cloning confirmed four specimens, collected from a robin (*Erithacus rubecula*) in Akerøya (cycle thresold: *Ct *= 30), a greenish warbler (*Phylloscopus trochiloides*) in Jomfruland (*Ct *= 24) and a bluethroat (*Luscinia svecica*) (*Ct *= 24) and a dunnock (*Prunella modularis*) from Store Færder (*Ct *= 27), to be positive. The disproportionality between *Babesia*-positive ticks caught from birds with an eastern versus a western migratory route was highly siginficant, i.e., *P*< 0.001 (Fisher's exact test for count data, run in the software R [28]).

**Figure 1:**
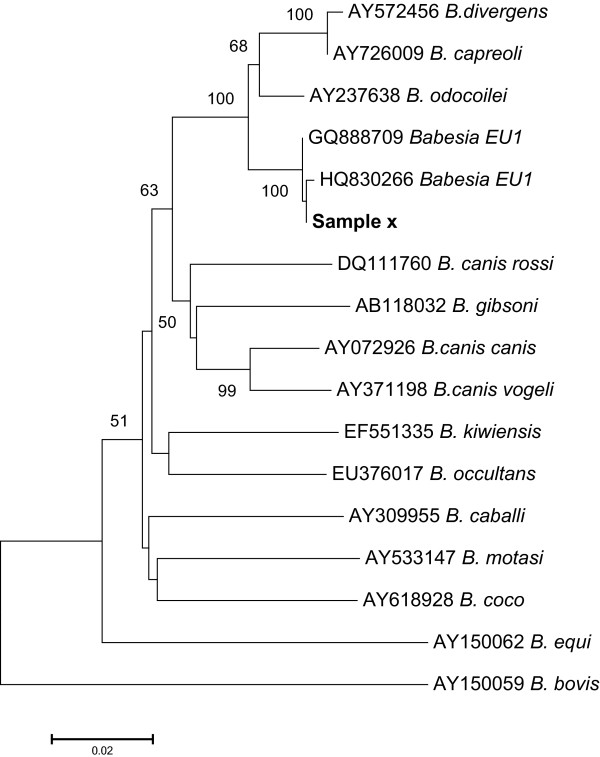
**Neighbour-joining tree of aligned 18S sequences after a bootstrap test with 500 replicates made using the Jukes-Cantor model **[33]. The tree is based on the final dataset that included 1,025 base pair positions (472+534) of the 18S *Babesia *sequences. The percentage of replicate trees, in which the associated taxa clustered together in the bootstrap test (500 replicates), are shown next to the branches (only values above 50 are displayed). Sequences from the unknown *Babesia *sample, 'Sample x', cluster with *B. venatorum *(EU1) sequences that were included in the alignment. Evolutionary analyses were conducted in MEGA 5 [27].

Phylogenetic analysis of the sequences obtained after cloning of the samples using a Neighbor-joining tree indicated a very high support for the unknown sequence to belong to *Babesia venatorum *(EU1) (GQ888709) (Bootstrap > 99) (Figure 1).

## Discussion

The real-time PCR used for screening was initially reported to be specific for *B. divergens *[17]. However, in this study we report the detection of *B. venatorum *using this method. In addition, the primer set and probe is capable of generating positive signals with DNA from other *Babesia *species, such as *B. canis canis *and *B. gibsoni *(Ø. Øines unpublished results). The fifth sample that was not confirmed to contain *Babesia *was only marginally positive in the real-time PCR, as it produced a signal close to the threshold line at 37 cycles, i.e., *Ct *= 36. This could indicate that either any *Babesia *DNA may have been present in this sample at concentrations that were too low to allow for verification using the procedures described previously, or, it could indicate that the cycle threshold (Ct) was set too high when selecting for positive samples. Hence positive signals would be false positives, due to unspecific products amplified in the real-time analysis.

Some bird species are partly migratory, with only a fraction of the individuals wintering in Norway [29]. Therefore, for a conservative approach, we only used engorged nymphs caught before or during the peak migratory arrival time in localities where ticks are endemic. In addition, three of the four bird species on which *B. venatorum *was found are not known to winter in Norway [29], and three *Babesia *samples were collected from birds on Akerøya and Store Færder (Table 1) where it seems to be no *I. ricinus *ticks [6]. Thus, it appears reasonable to conclude that the ticks infected by *B. venatorum *were brought to Norway by the birds they were feeding on. The low number of *Babesia*-positive nymphs was insufficient for analyses comparing differences in the prevalence of these nymphs by bird species. The robin and dunnock belong to the great majority of birds that migrate to Norway via the Atantic coast of continental Europe [29]. The greenish warbler does not breed west of Gothland, Sweden [30] and is very rarely encountered in Norway, and the bluethroat is one of the few birds that migrate to Norway via an eastern migratory route [29]. Thus, two of the four *B. venatorum*-positive ticks were collected from the minority of birds that came from Eastern Europe, indicating that *B. venatorum *may be more common in Eastern Europe.

**Table 1:** Number of positive (percentage) *Babesia venatorum *(EU 1) specimens detected by PCR of *Ixodes ricinus *nymphs collected from northward migrating birds

Bird observatory	*Babesia venatorum*	Total ticks
Akerøya	1 (0.6)	175
Jomfruland	1 (1.1)	93
Lista	0	80
Store Færder	2 (1.2)	164
Total positive	4 (0.8)	512

In parentheses: per cent.

*B. venatorum (Babesia *EU1) had previously not been reported in Norway. Thus, we did not expect to find this parasite and not *B. divergens*, which is a widespread and common parasite of cattle [12,15]. Duh et al. found *B. venatorum *in 11 and *B. divergens *in 28 of 51 roe deers that were tested [19]. The host range for *B. venatorum *is poorly understood, and apart from splenectomised humans, a literature search on the Internet (http://www.scholar.google.com) revealed no natural hosts besides roe deer or any other tick vector than *I. ricinus *for *B. venatorum*.

Few *B. venatorum*-positive ticks were found on migratory birds in this study. There are 30-85 million passerine birds that migrate to Norway every spring [31], which may carry 0.2 ticks on average (nymphs and larvae) across the sea during their spring migration [6]. *B. divergens *has been shown to be transmitted transovarially and transstadially in the tick [32]. Therefore, larvae are as likely as nymphs to carry the pathogen. Assuming a prevalence of infection less than 1%, as was obtained in this study, birds could introduce on the order of 100,000 *Babesia*-infected ticks per year. Therefore, the spring migration represents an important component in the mechanism of the spread of tick-borne pathogens. Although theoretically possible, birds have not been shown to be responsible for the introduction of new tick-borne pathogens. This study shows that such introduction of *B. venatorum *is possible. Evidence suggests that the transport of ticks by migratory birds into Norway has increased over the last four decades [6], and it is possibile that *B. canis*, recently described for the first time in Norway [11], may have been introduced by ticks on migratory birds.

## Competing interests

The authors declare that they have no competing interests.

## Authors' contributions

GH organised the fieldwork and wrote the main part of the paper. KHR led the DNA-isolation part of the laboratory work. ØØ performed the nested PCR, cloning and phylogeny of the positive samples from the real-time PCR screening. ØØ, KHR and HPL provided valuable and significant contributions to the writing of the paper. All authors read and approved the final manuscript.

## References

[B1] Korch GW Geographic dissemination of tick-borne zoonoses 1994 New York, Oxford: Oxford University Press

[B2] Hoogstraal H Kaiser MN Traylor MA Gaber S Guindy E Ticks (Ixodoidea) on birds migrating from Africa to Europe and Asia Bull World Health Organ 1961 24 197 212 13715709 PMC2555510

[B3] Mehl R Michaelsen J Lid G Ticks (Acari, Ixodides) on migratory birds in Norway Fauna Norv Ser B 1984 31 46 58

[B4] Olsén B Jaenson T Bergstrom S Prevalence of *Borrelia burgdorferi *sensu lato-infected ticks on migrating birds Applied and Environmental Microbiology 1995 61 3082 7487041 10.1128/aem.61.8.3082-3087.1995 PMC167585

[B5] Poupon M Lommano E Humair P Douet V Rais O Schaad M Jenni L Gern L Prevalence of *Borrelia burgdorferi *sensu lato in ticks collected from migratory birds in Switzerland Applied and Environmental Microbiology 2006 72 976 10.1128/AEM.72.1.976-979.2006 16391149 PMC1352204

[B6] Hasle G Bjune G Edvardsen E Jakobsen C Linnehol B Røer J Mehl R Røed K Pedersen J Leinas H Transport of ticks by migratory passerine birds to Norway J Parasitol 2009 95 1342 1351 10.1645/GE-2146.1 19658452

[B7] Estrada-Peña A Jongejan F Ticks feeding on humans: a review of records on human-biting Ixodoidea with special reference to pathogen transmission Experimental and Applied Acarology 1999 23 685 715 10.1023/A:1006241108739 10581710

[B8] Gorenflot A Moubri K Precigout E Carcy B Schetters TP Human babesiosis Annals of Tropical Medicine and Parasitology 1998 92 489 501 10.1080/00034989859465 9683900

[B9] Levine N Taxonomy of the piroplasms Transactions of the American Microscopical Society 1971 90 2 33 10.2307/3224894

[B10] Peirce M A taxonomic review of avian piroplasms of the genus *Babesia Starcovici*, 1893 (Apicomplexa: Piroplasmorida: Babesiidae) Journal of Natural History 2000 34 317 332 10.1080/002229300299507

[B11] Øines Ø Storli K Brun-Hansen H First case of babesiosis caused by *Babesia canis canis *in a dog from Norway Vet Parasitol 2010 171 350 353 10.1016/j.vetpar.2010.03.024 20378251

[B12] Zintl A Mulcahy G Skerrett HE Taylor SM Gray JS *Babesia divergens*, a bovine blood parasite of veterinary and zoonotic importance Clin Microbiol Rev 2003 16 622 636 10.1128/CMR.16.4.622-636.2003 14557289 PMC207107

[B13] Stuen S *Anaplasma phagocytophilum *(formerly *Ehrlichia phagocytophila*) infection in sheep and wild ruminants in Norway 2002 Oslo: Norwegian School of Veterinary Science

[B14] Thambs-Lyche H *Ixodes ricinus *og piroplasmosen I Norge Norsk Veterinærtidsskrift 1943 60 337 366

[B15] Hasle G Bjune GA Christensson D Roed KH Whist AC Leinaas HP Detection of *Babesia divergens *in southern Norway by using an immunofluorescence antibody test in cow sera Acta Vet Scand 2010 52 55 10.1186/1751-0147-52-55 20925923 PMC2959048

[B16] Lundsett AL Flåtten *Ixodes ricinus *som sykdomsvektor i Sør-Norge MSc dissertation 2004 Telemark University College

[B17] Radzijevskaja J Paulauskas A Rosef O Prevalence of *Anaplasma phagocytophilum *and *Babesia divergens *in *Ixodes ricinus *ticks from Lithuania and Norway Int J Med Microbiol 2008 298 218 221

[B18] Herwaldt BL Cacciò S Gherlinzoni F Aspöck H Slemenda SB Piccaluga PP Martinelli G Edelhofer R Hollenstein U Poletti G Molecular characterization of a non-Babesia divergens organism causing zoonotic babesiosis in Europe Emerging Infectious Diseases 2003 9 943 10.3201/eid0908.020748 PMC3020600 12967491

[B19] Duh D Petrovec M Bidovec A Avsic-Zupanc T Cervids as babesiae hosts, Slovenia Shouxi 2005 11 10.3201/eid1107.040724 PMC3371785 16022795

[B20] Bonnet S Jouglin M L'Hostis M Chauvin A *Babesia *sp. EU1 from Roe Deer and Transmission within *Ixodes ricinus* Emerging Infectious Diseases 2007 13 10.3201/eid1308.061560 PMC2828078 17953093

[B21] Duh D Petrovec M Avsic-Zupanc T Molecular characterization of human pathogen Babesia EU1 in Ixodes ricinus ticks from Slovenia Journal of Parasitology 2005 91 463 465 10.1645/GE-394R 15986627

[B22] Casati S Sager H Gern L Piffaretti J Presence of potentially pathogenic Babesia sp. for human in Ixodes ricinus in Switzerland Annals of agricultural and environmental medicine 2006 13 65 70 16841874

[B23] Häselbarth K Tenter AM Brade V Krieger G Hunfeld KP First case of human babesiosis in Germany-Clinical presentation and molecular characterisation of the pathogen Int J Med Microbiol 2007 297 197 204 10.1016/j.ijmm.2007.01.002 17350888

[B24] Skotarczak B Rymaszewska A Wodecka B Sawczuk M Adamska M Maciejewska A PCR detection of granulocytic Anaplasma and Babesia in Ixodes ricinus ticks and birds in west-central Poland Annals of agricultural and environmental medicine 2006 13 21 23 16841867

[B25] Gray J Stanek G Kundi M Kocianova E Dimensions of engorging *Ixodes ricinus *as a measure of feeding duration Int J Med Microbiol 2005 295 567 572 10.1016/j.ijmm.2005.05.008 16325552

[B26] Zintl A Finnerty EJ Murphy TM de Waal T Gray JS Babesias of red deer (Cervus elaphus) in Ireland Vet Res 2011 42 10.1186/1297-9716-42-7 PMC3037898 21314977

[B27] Tamura K Peterson D Peterson N Stecher G Nei M Kumar S MEGA5: Molecular Evolutionary Genetics Analysis using Maximum Likelihood, Evolutionary Distance, and Maximum Parsimony Methods. Molecular Biology and Evolution Molecular Biology and Evolution 2011 10.1093/molbev/msr121 PMC3203626 21546353

[B28] R: A language and environment for statistical computing http://www.R-project.org

[B29] Bakken V Runde O Tjørve E Norwegian bird ringing atlas 2006 Stavanger: Stavanger Museum

[B30] Svensson L Grant PJ Mullarney K Zetterström D Fågelguiden 1999 Stockholm: Bonniers förlag

[B31] Gjershaug JO Thingstad PG Eldøy S Byrkjeland S *Norsk fugleatlas *Klæbu 1994 Norway: Norsk Ornitologisk Forening

[B32] Bonnet S Jouglin M Malandrin L Becker C Agoulon A L'Hostis M Chauvin A Transstadial and transovarial persistence of *Babesia divergens *DNA in *Ixodes ricinus *ticks fed on infected blood in a new skin-feeding technique Parasitology 2007 134 197 207 10.1017/S0031182006001545 17076925

[B33] Jukes TH Cantor CR Evolution of protein molecules 1969 New York: Academic Press

